# Double-Strand Break Repair by Interchromosomal Recombination: An *In Vivo* Repair Mechanism Utilized by Multiple Somatic Tissues in Mammals

**DOI:** 10.1371/journal.pone.0084379

**Published:** 2013-12-13

**Authors:** Ryan R. White, Patricia Sung, C. Greer Vestal, Gregory Benedetto, Noelle Cornelio, Christine Richardson

**Affiliations:** 1 Department of Biology, University of North Carolina-Charlotte, Charlotte, North Carolina, United States of America; 2 Developmental Biology, Sloan-Kettering Institute, Memorial Sloan-Kettering Cancer Center, New York, New York, United States of America; National Cancer Institute, United States of America

## Abstract

Homologous recombination (HR) is essential for accurate genome duplication and maintenance of genome stability. In eukaryotes, chromosomal double strand breaks (DSBs) are central to HR during specialized developmental programs of meiosis and antigen receptor gene rearrangements, and form at unusual DNA structures and stalled replication forks. DSBs also result from exposure to ionizing radiation, reactive oxygen species, some anti-cancer agents, or inhibitors of topoisomerase II. Literature predicts that repair of such breaks normally will occur by non-homologous end-joining (in G1), intrachromosomal HR (all phases), or sister chromatid HR (in S/G^2^). However, no *in vivo* model is in place to directly determine the potential for DSB repair in somatic cells of mammals to occur by HR between repeated sequences on heterologs (i.e., interchromosomal HR). To test this, we developed a mouse model with three transgenes—two nonfunctional green fluorescent protein (GFP) transgenes each containing a recognition site for the I-*Sce*I endonuclease, and a tetracycline-inducible I-*Sce*I endonuclease transgene. If interchromosomal HR can be utilized for DSB repair in somatic cells, then I-*Sce*I expression and induction of DSBs within the GFP reporters may result in a functional GFP+ gene. Strikingly, GFP+ recombinant cells were observed in multiple organs with highest numbers in thymus, kidney, and lung. Additionally, bone marrow cultures demonstrated interchromosomal HR within multiple hematopoietic subpopulations including multi-lineage colony forming unit–granulocyte-erythrocyte-monocyte-megakaryocte (CFU-GEMM) colonies. This is a direct demonstration that somatic cells *in vivo* search genome-wide for homologous sequences suitable for DSB repair, and this type of repair can occur within early developmental populations capable of multi-lineage differentiation.

## Introduction

Faithful repair of DNA damage, including double-strand breaks (DSBs), is crucial to genome stability and normal cell survival and proliferation [1]. Chromosomal breaks can occur in a programmed manner through meiosis, immunoglobulin class-switch recombination, and V(D)J recombination [2–4]. In addition, reactive oxidative species may promote 10,000-20,000 DNA damaged sites per cell per day [5–7], and DNA replication errors or stalls may promote another 10-50 DSBs per cell [8,9]. Exposure to ionizing radiation (IR), alkylating agents, and chemotherapeutic drugs such as topoisomerase II inhibitors also promote chromosomal breaks [10–14]. Some environmental and/or dietary compounds may promote DSBs, and the recent observations that bioflavonoids can stabilize DNA DSBs and lead to illegitimate repair and genome rearrangements in cultured cells underscores the importance of understanding DSB repair processes *in vivo* [15–18]. 

DSBs are potent inducers of recombination and increase both homologous recombination (HR) and non-homologous end-joining (EJ) events by several orders of magnitude [19,20]. These two major DSB repair pathways differ based on their requirement for a donor DNA template with significant sequence homology; thus, their relative activity changes with each stage of the cell cycle. Studies in multiple organisms have demonstrated that EJ is most efficient in G1 and in noncycling somatic cells while homology-directed DSB repair is favored in both S/G^2^ utilizing a sister chromatid and intrachromosomal HR [19,21–26]. *In vivo* systems have been developed to detect EJ, sister chromatid, and intrachromosomal HR that arise both spontaneously and in response to induced DSBs [27–30]. Homologs are utilized for HR-directed DSB repair with lower efficiency although this is increased in organisms that exhibit a high degree of mitotic pairing, supporting the hypothesis that proximity of homologous sequences is an important factor in determining template choice [31–33]. While repair of specific DSBs by more distant homologous repeat sequences on heterologous chromosomes (i.e. interchromosomal HR) has been examined *in vivo* using mitotic yeast and tobacco [34,35], studies in mammalian cells have been limited to cultured cell assays [36–39]. Whether repair of DSBs *in vivo* in mammals occurs by interchromosomal HR at significant and detectable frequencies has not been demonstrated.

If cells are exposed to irradiation, chemotherapeutic agents, or even environmental factors and metabolites, multiple DSBs at unlinked loci will occur in the same cell at the same time. Repair of multiple breaks using interchromosomal HR *in vivo* has the potential to result in reciprocal exchanges that may be viable, inherited by daughter cells in the next cell division, or inherited through the germ line. Genome analysis of plants suggests that translocations are a regular mechanism of plant evolution [40,41]. In mammals, one third of the genome is composed of repetitive elements [42]. The presence of Alu elements elevates recombination rates [43], and Alu-Alu mediated recombination has been associated with founder mutations and evolution [44–49]. In somatic cells, translocations can be tumorigenic, and are a hallmark of human hematopoietic malignancies and some soft-tissue sarcomas [36,50–56]. Thus, such events would likely be suppressed in somatic cells *in vivo* where a selective pressure exists to maintain genome stability and avoid immortalization. Specialized cell types within mammals may preferentially utilize different pathways of repair, particularly as more differentiated cells spend less time in S phase of the cell cycle [57–60] or as proliferation rates change with age [61,62].

To directly test the potential for multiple DSBs to promote interchromosomal HR *in vivo* in mammals, we developed a mouse model with three transgenes--two nonfunctional green fluorescent protein (GFP) reporter transgenes each containing a recognition site for the I-*Sce*I endonuclease, and a tetracycline-inducible I-*Sce*I endonuclease transgene. Induced expression of I-*Sce*I and the resulting induction of DSBs within the GFP reporters may produce a functional GFP gene if interchromosomal HR is utilized for repair. In this system, GFP+ recombinant cells were observed in all seven organs examined--pancreas, liver, spleen, kidney, thymus, heart, and lung--with highest numbers in thymus, kidney, and lung. Bone marrow cultures demonstrated interchromosomal HR within multiple colony types including early progenitor CFU-GEMM. This is a direct demonstration that somatic cells *in vivo* maintain the potential to search genome-wide for homologous sequences suitable for DSB repair, and this type of repair can occur within progenitor populations capable of proliferation and multi-lineage differentiation. 

## Results

### *In vivo* mouse model

**Figure 1:**
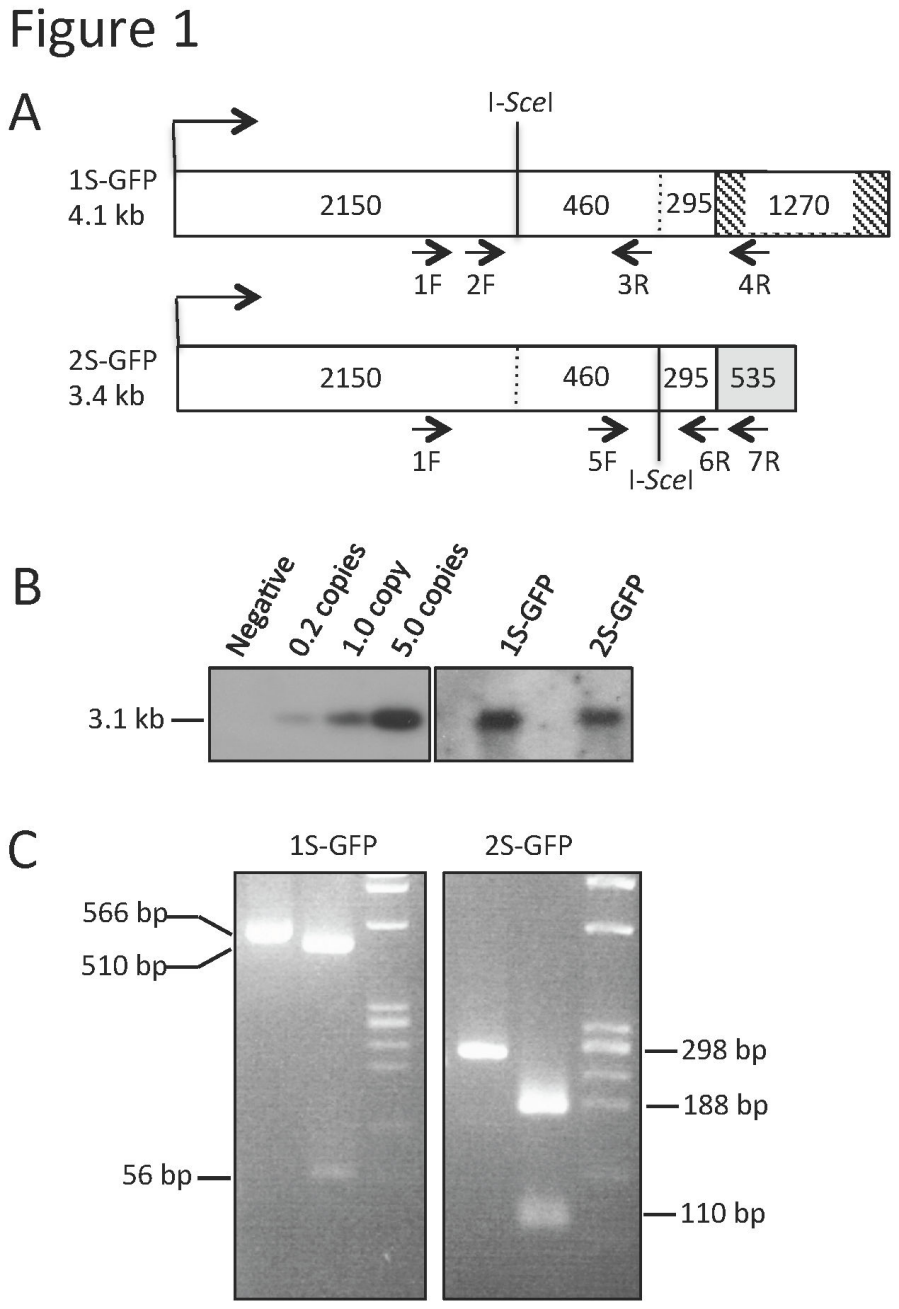
Structure and confirmation of the 1S and 2S GFP transgenes. (**A**) For each construct schematic, the numbers of bases are indicated to show the lengths of homology between the two as well as the relative positions of the engineered I-*Sce*I restriction sites. The 3’UTR sequences of the two constructs do not share homology and are indicated as a hatched box of 1270 bp for 1S-GFP and a grey box of 535 bp for 2S-GFP; these non-homologous sequences allow for PCR amplification specific to each transgene. Nested PCR primer pairs used for verification of intact construct sequences and for analysis of GFP+ hematopoietic colonies are indicated. Primers 1F-4R followed by 2F-3R amplify sequence flanking the I-*Sce*I site in 1S-GFP. Primers 1F-7R followed by 5F-6R amplify sequence flanking the I-*Sce*I site in 2S-GFP. (**B**) Southern blotting to estimate copy number utilized a GFP ORF DNA fragment of 3.1 kb and diluted to pg amounts that approximated 0, 0.2, 1.0, and 5.0 copies per genome spiked into 10µg non-transgenic mouse DNA. Genomic DNA from single transgenic mice (either 1S-GFP or 2S-GFP) was digested with restriction endonucleases within the GFP promoter and ORF of both transgenes to yield a 3.1 kb fragment. Band intensities are consistent with 4-5 copies of 1S-GFP and 2-4 copies of 2S-GFP, and were confirmed with Q-PCR data on the same samples (data not shown). (**C**) PCR reactions flanking each DSB site in the two GFP constructs confirm intact I-SceI recognition sites. Nested PCR as described in Materials amplified each transgene shown in the left side lane of each image. Digestion with I-*Sce*I endonuclease produced the expected sizes indicated in the middle lane of each image. Right side Marker lane PhiX.

**Figure 2:**
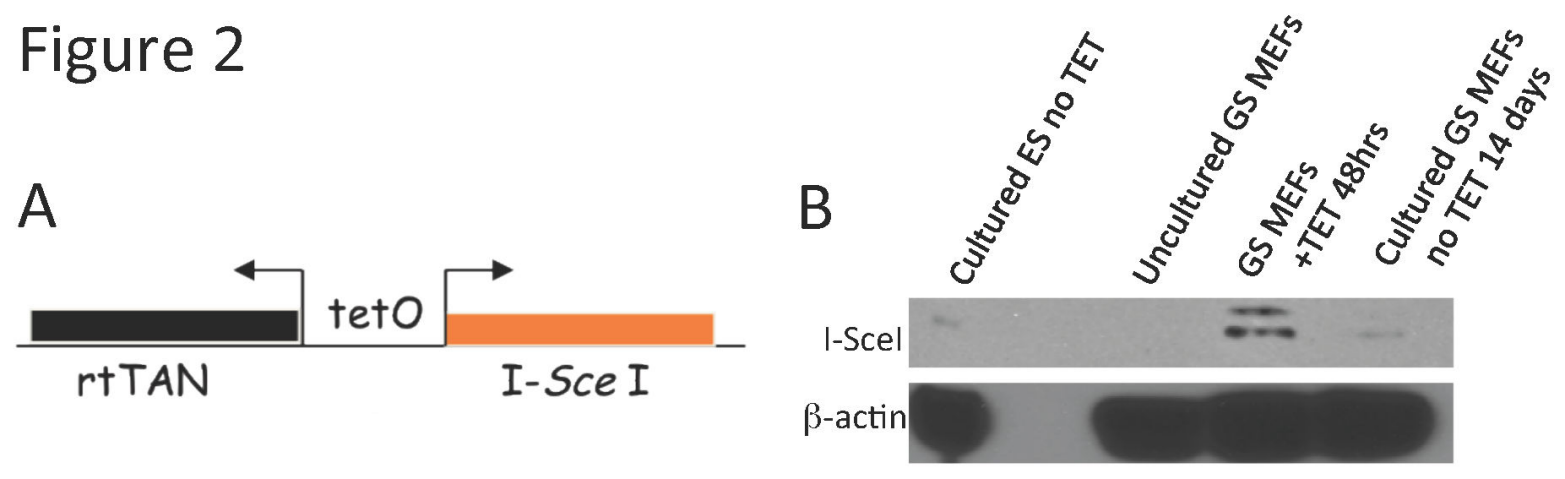
Structure and confirmation of the tetracycline inducible I-*Sce*I transgene. (**A**) For details of the bicistronic I-*Sce*I transgene construct refer to [65]. (**B**) MEFs derived from GS mice were cultured in media supplemented with TET at 2μg/mL for 48 hours. Total protein extracts were harvested and analyzed by Western blotting. By 48 hours post-TET, detectable quantities of I-*Sce*I endonuclease can be observed. As a negative controls, total protein extracts were harvested from cultured E14 ES cells or uncultured MEFs from GS mice. Loading control: Western blotting for β-actin.

 Constructs were designed to introduce two defective green fluorescent protein (GFP) genes and a tetracycline-responsive (TET-ON) inducible I-*Sce*I endonuclease gene construct onto heterologous chromosomes in the mouse genome. 1S-GFP and 2S-GFP reporter constructs each contain a unique 18bp restriction site for the endonuclease I-*Sce*I [63,64] in the 5’ and 3’ ORF regions, respectively, with 460bp homology to each other between the two restriction sites (Figure 1A). The TET-ON I-*Sce*I endonuclease gene is on a single auto-regulated bi-directional expression vector with the tet operator regulating both a TK-rtTAN repressor of the transactivator gene (vector kindly provided by Craig Strathdee) [65] and an I-*Sce*I gene (Figure 2A) [64,66]. Presence of the transgenes within mice was shown by both Southern Blotting and PCR of DNA isolated from tail tips. Founder mice containing each transgene were crossed with wild type, and those that inherited single insertion sites at Mendelian ratios and with the lowest copy number as estimated by both Southern blotting and Q-PCR as compared against a standard (Figure 1B and Methods) were maintained for further breeding. Taken together these analyses estimated 4-5 copies of 1S-GFP and 2-4 copies of 2S-GFP. Mice were screened for an intact I-*Sce*I site at both the 1S-GFP and the 2S-GFP reporters using PCR primers that flank each I-*Sce*I site and digestion of the PCR product with I-*Sce*I endonuclease (Figure 1A, 1C). Individually 1S-GFP and 2S-GFP positive lines were crossed to each other, and then crossed to the I-*Sce*I transgenic line over generations, and inheritance of the three transgenes in expected Mendelian ratios supports unlinked loci. Breeding resulted in triply positive transgenic GS lines for analysis.

### DSB-induced interchromosomal HR occurs in mouse embryonic fibroblasts

 Mouse embryonic fibroblasts (MEFs) were harvested at day E13.5. MEFs from each GS mouse were divided and cultured in one of 3 conditions: (1) cultured in media without DSB induction, (2) cultured in the presence of tetracycline (2 μg/mL) to induce DSBs through I-*Sce*I expression, or (3) transfected with 30μg I-*Sce*I expression vector CBAS [20] to induce DSBs through I-*Sce*I expression. I-*Sce*I RNA transcripts and protein were detectable by RT-PCR and Western blotting, respectively, following addition of tetracycline to culture media of MEFs (Figure 2B) or to H_2_O provided transgenic mice in subsequent experiments (see below).

**Figure 3:**
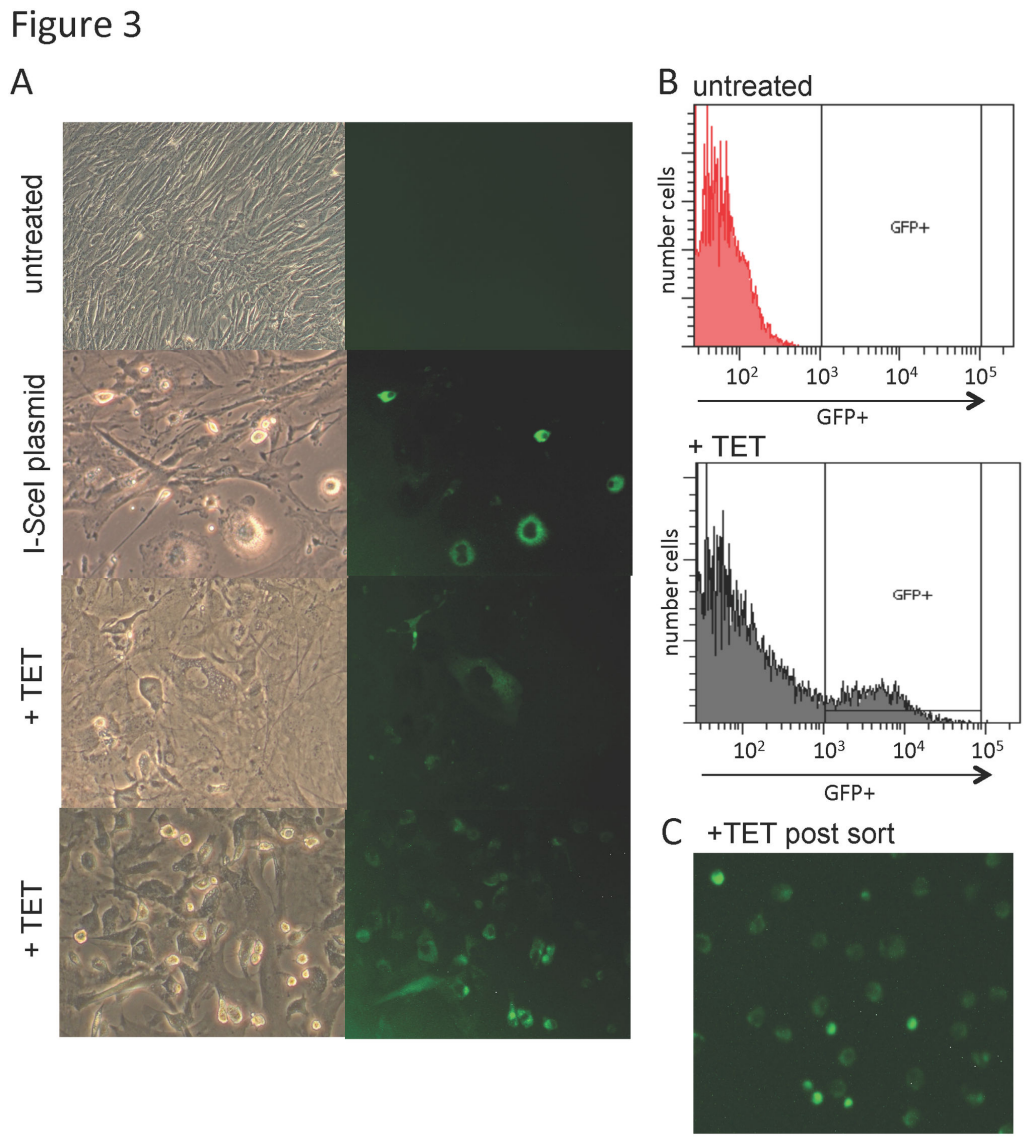
Analysis of GFP+ MEFs post-TET. (**A**) Phase contrast and matched fluorescent microscopy images of MEFs in culture—magnification 400X. Top row-untreated MEFs. Second row-96 hrs post-electroporation with I-*Sce*I expression plasmid. Third and fourth rows-96 hrs after addition of tetracycline to the culture medium (+TET). (**B**) Representative FACS plot of MEFs with GFP positivity in log scale on the x axis plotted against number of cells on the y axis. Upper plot--untreated MEFs. Lower plot-- +TET treated MEFs. In this sample, the GFP+ population is 12.4%. (**C**) Confirmation of GFP+ cells after FACS single cell sorting for GFP+ MEFs. Cells within the GFP+ gate indicated in B lower panel were sorted and then viewed by fluorescent microscopy—magnification 400X.

Individual GFP+ MEFs were detectable by inverted fluorescent microscopy as early as 4 days following the addition of tetracycline (Figure 3A). Cells were analyzed by fluorescent activated cell sorting (FACS) 6-10 days post-tetracycline. Untreated MEFs had an undetectable number of GFP+ cells. By contrast, intermediate/bright GFP+ cells were greater than 12% of the treated cells (compared against untreated cells with a gate set at 0.1%; n=12) (Figure 3B). Individual GFP+ cells were FACS sorted and confirmed to be GFP+ by inverted fluorescent microscopy (Figure 3C).

### DSB-induced interchromosomal HR occurs *in vivo* in multiple somatic cell types

**Figure 4:**
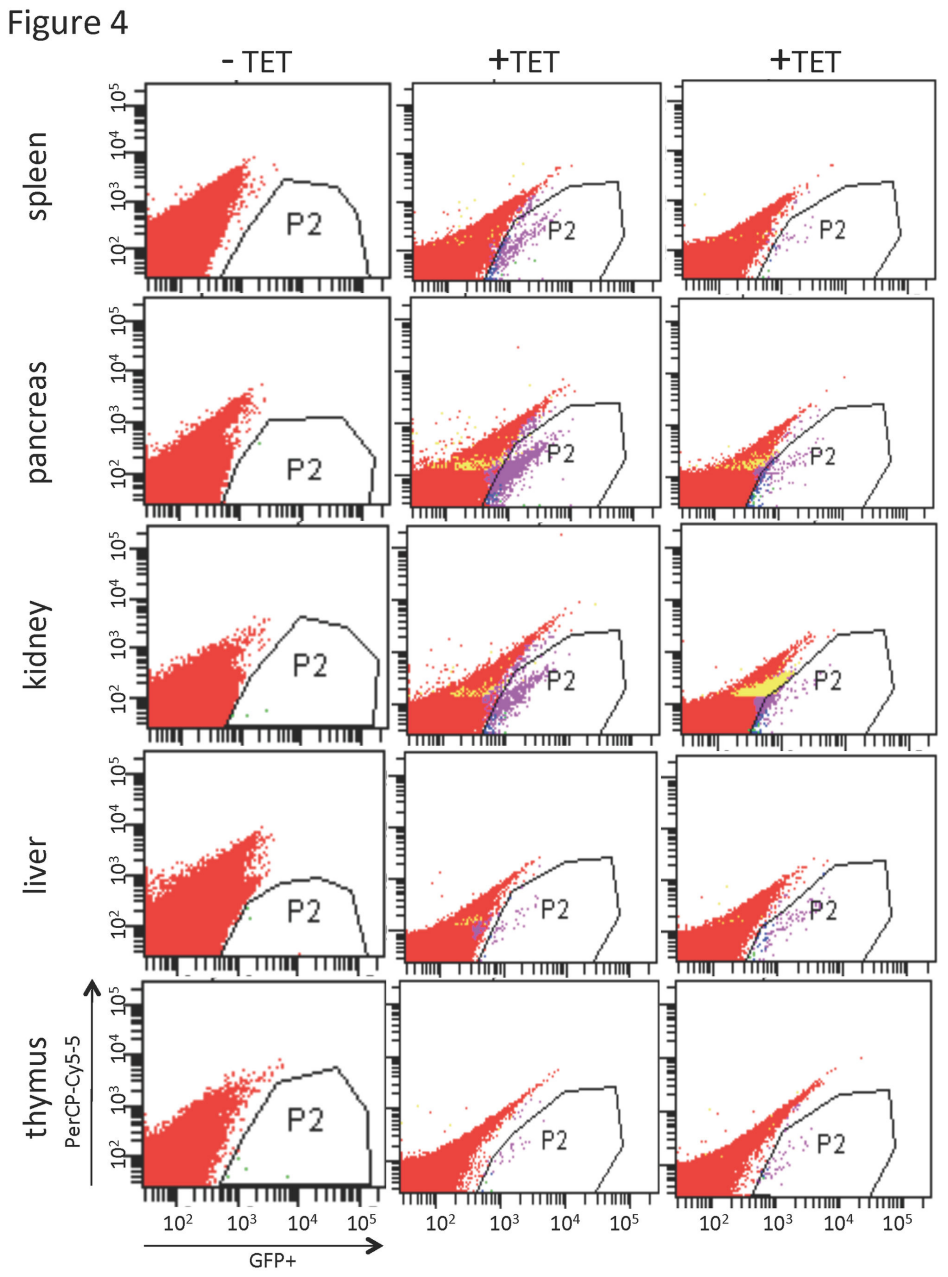
Representative FACS analysis plots of three GS mice for spleen, pancreas, kidney, liver, and thymus. GFP positivity is shown on log scale on the X axis plotted against nonspecific PerCP-Cy5-5 on the Y axis to visualize individual cells. Age-matched negative control mice were not provided TET (-TET). Two representative age-matched mice contain all 3 transgenes and were provided TET as described in text (+TET). Establishment of gates is described in text.

**Figure 5:**
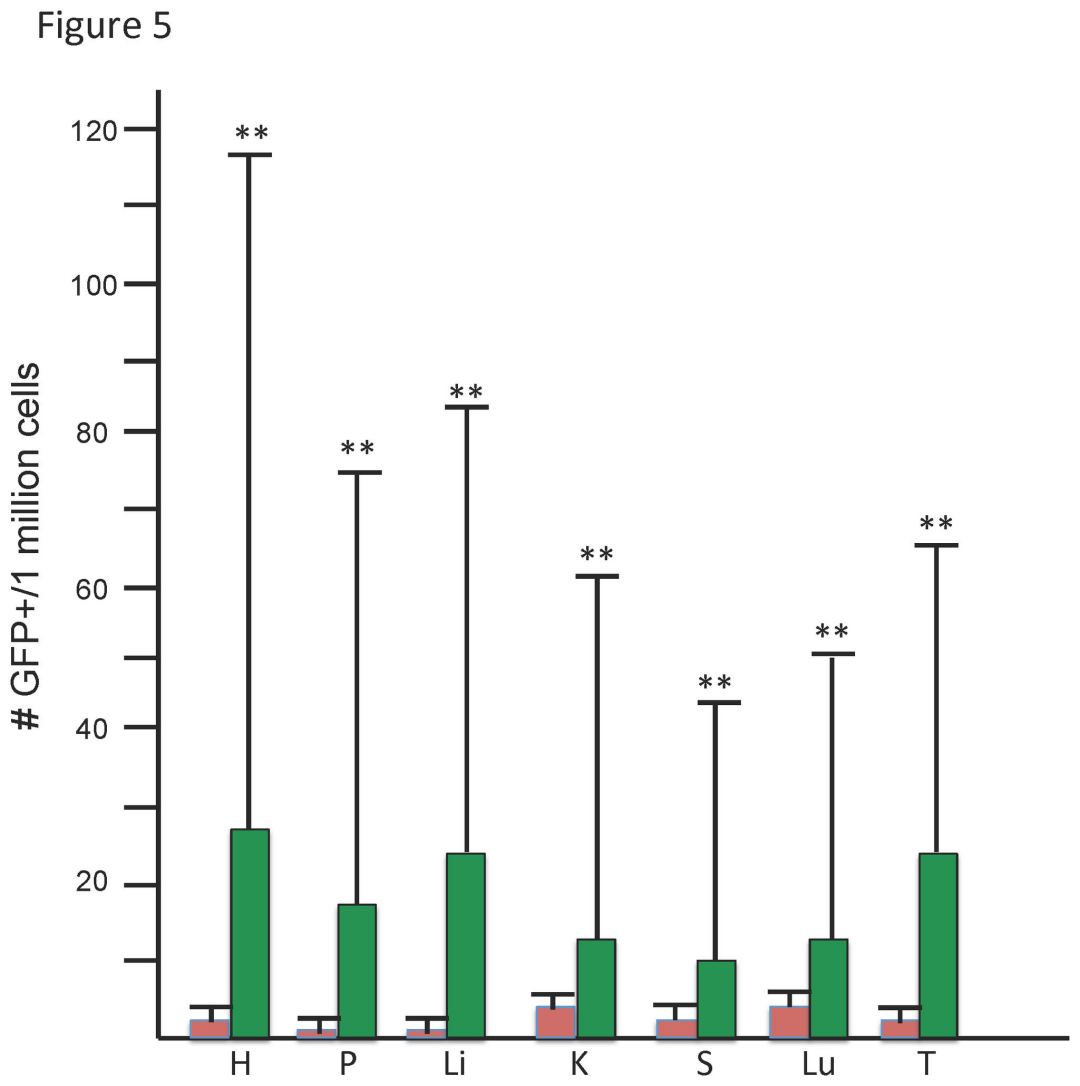
Quantitative analysis of GFP+ cells in all mice analyzed. The number of GFP+ cells in each organ of analyzed mice was determined. Establishment of gates is described in text. From FACS analysis, the average number of GFP+ recombinant cells per million cells and the standard deviation of each was calculated for seven organs and represented in bar graph form. Negative controls are shown in red bars (n=8), and +TET are shown in green bars (n=47). H=heart, P=pancreas, Li=liver, K=kidney, S=spleen, Lu=lung, T=thymus. Organs with statistically significant increased numbers of GFP+ cells groups are indicated by ** above the error bars.

 GS mice at least 3 months of age (n=47) were administered tetracycline through H_2_0 for 21d to allow an extended period of I-*Sce*I expression and subsequent induction of DSBs. Mice were then taken off tetracycline for 7d-21d prior to analysis. This waiting period would restrict analysis to viable GFP+ cells after cells with unstable repair structures would be cleared from the *in vivo* tissues. A total of seven organs--pancreas, liver, spleen, kidney, thymus, heart, and lung--were analyzed for GFP+ recombinants by FACS (Figure 4). Mice were analyzed in batches, and each batch included an age-matched non-transgenic mouse (n=8). Gates for determination of GFP+ cells were set such that negative controls had ≤3 events per million, and then the same gates were used to score GFP+ cells from GS tetracycline-treated mice. This analysis directly demonstrated that GFP+ cells, as determined by >3 GFP+ cells per million by FACS, were readily detectable in multiple tissues from 40 of the 47 mice treated and analyzed (Figures 4,5; Table S1). Despite variance in GFP+ numbers detected between mice, all organs had significantly increased GFP+ cells as compared to the age-matched negative controls (Figure 5). For comparison, constitutively expressing EGFP mice consistently contained >45% GFP+ cells in all tissues examined (data not shown) [67]. These data demonstrate that somatic cell types *in vivo* retain the potential to repair DSBs with a homologous sequence on a heterologous chromosome. Furthermore, the potential for interaction between sequences on heterologous chromosomes in wild-type cells has not been eliminated by epigenetic factors or chromatin remodeling associated with differentiation programs.

 Additionally, age-matched GS mice that were not administered tetracycline were analyzed (n=15). 13 of 15 of these mice had undetectable levels of GFP+ cells in all organs examined, similar to the non-transgenic controls. However, two of the 15 mice contained GFP+ populations of cells in multiple tissues (data not shown). In these mice, it is possible that the I-*Sce*I transgene became activated. Alternatively, it is possible that an early progenitor cell *in utero* underwent spontaneous interchromosomal HR giving rise to a GFP+ progenitor cell that contributed to multiple tissues, or was a cell type that gave rise to cells capable of infiltrating multiple organs, e.g. circulating hematopoietic cells.

### Impact of aging on DSB-induced interchromosomal HR in multiple somatic cell types

**Figure 6:**
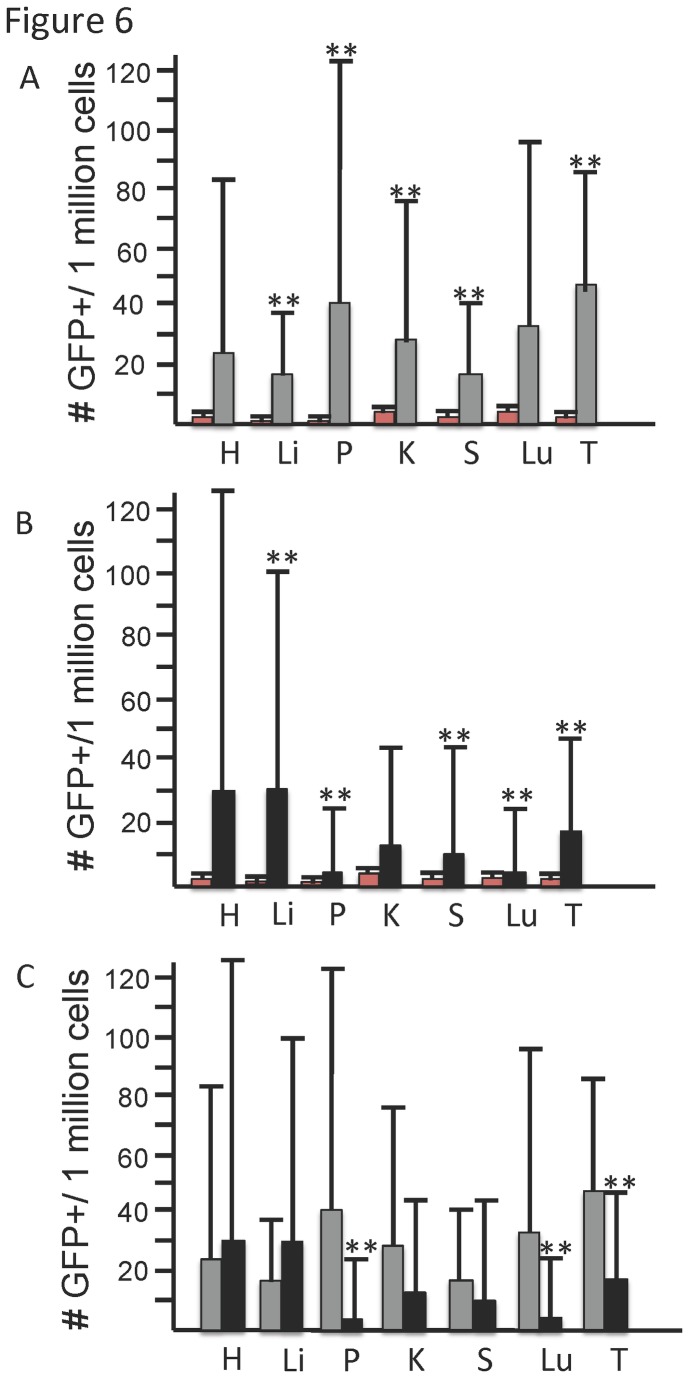
Quantitative analysis of GFP+ cells by age. (**A**) In the young mouse cohort (age<5.5 months), negative controls are shown in red bars (n=5), and +TET are shown in grey bars (n=16). Organs with statistically significant increased numbers of GFP+ cells groups are indicated by ** above the error bars. (**B**) In the older mouse cohort (age>8 months), negative controls are shown in red bars (n=11) and +TET are shown in black bars (n=31). (**C**) Comparison of +TET young mice (grey bars) versus +TET old mice (black bars) from A and B. Organs with statistically significant different numbers of GFP+ cells by age are indicated by ** above the error bars. For all panels, H=heart, P=pancreas, Li=liver, K=kidney, S=spleen, Lu=lung, T=thymus.

 Close examination of the variance of numbers of GFP+ cells detected in tetracycline-treated GS mice indicated that 7 of the 47 mice contained no detectable GFP+ cells in any organs analyzed, similar to non-transgenic controls. All 7 mice were older. Thus, we separated analysis of the 47 mice GS mice into two age cohorts, young (≤ 5months old, n=16) and old (≥ 8 months, n=31) (Figures 6A and 6B, respectively). Regardless of age, statistically significant numbers of GFP+ cells were in most organs examined, as compared to negative control mice. Comparison of GFP+ cell numbers by age (Figure 6C) indicated that in 5 of the 7 organs examined (pancreas, kidney, spleen, lung, and thymus), overall numbers of detectable GFP+ cells were lower in the cohort of older mice (Figure 6C). The decrease in detectable number of GFP+ cells was significant in 3 of these (pancreas, lung, thymus). However, two organs (heart and liver) appeared to have an overall slight increase in numbers GFP+ cells in older mice, although the trend did not reach statistical significance. Decreases in transgene expression levels with age has been observed in multiple other models. A similar mechanism of transgene shutdown may be involved in this model, but only occur in a subset of tissue types. It is possible that certain organs contain specific cell types or progenitor cells capable of DSB-induced interchromosomal HR, even within older mice. Further determination of the specific cell types that are GFP+ within each of the mice could provide this information. 

Given the variance in GFP+ numbers detected between mice (Figures 5, 6; Table S1), statistical significance of the probabilities associated with covariance among the traits was calculated for each pair of traits separately in the young and old cohorts (Table S2). In the young cohort, only a single strong positive correlation of covariance between spleen and kidney (p=0.002) was noted. In the old cohort, a larger number, although weaker, of positive correlations of covariance were noted; these were between heart and pancreas (p=0.021) or thymus (0.021), as well as between spleen and kidney (0.021) or thymus (p=0.021). 

### DSB-induced interchromosomal HR occurs *in vivo* in hematopoietic multi-lineage progenitor cell types

**Figure 7:**
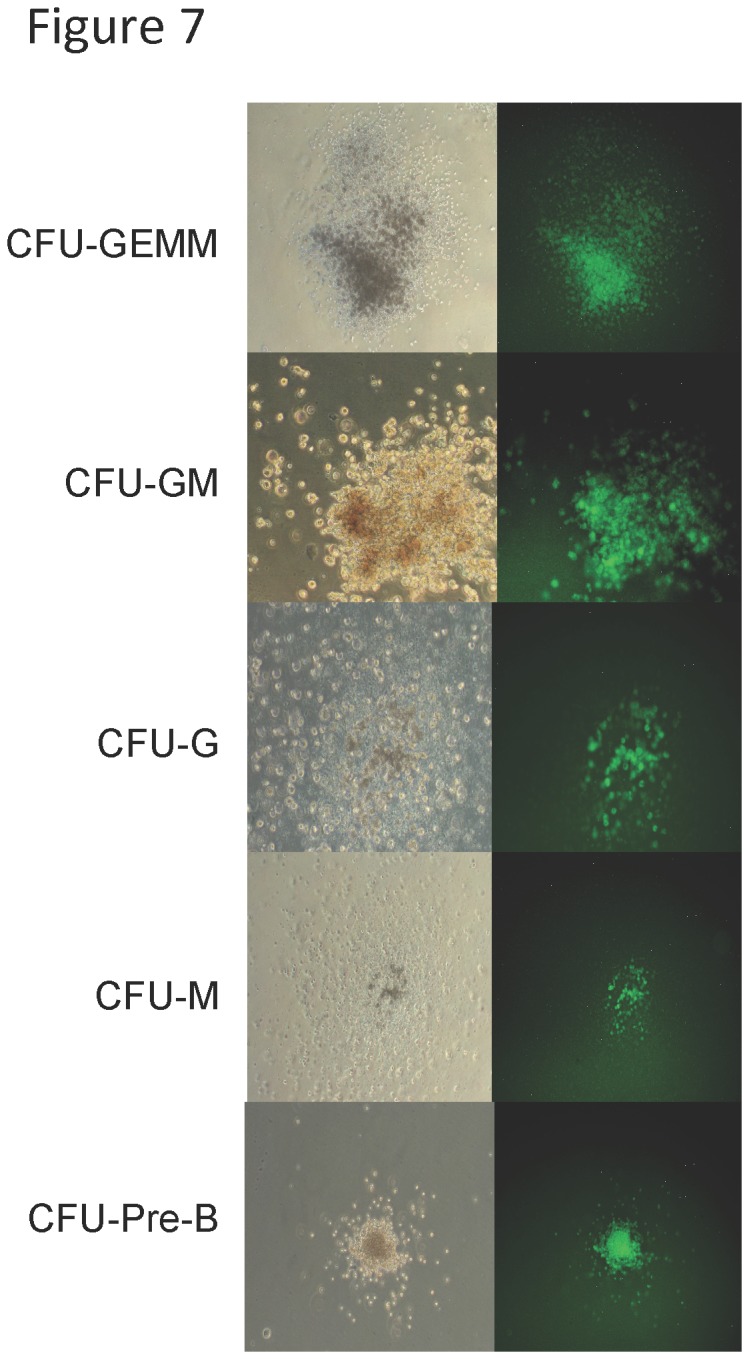
DSB-induced GFP+ recombinants in hematopoietic subpopulations isolated from bone marrow of GS mice. Colonies were scored by inverted fluorescent microscopy and faint background fluorescence of negative controls was subtracted out of total repair frequency. Representative phase contrast and fluorescent microscopy images of GFP+ recombinants from bone marrow CFC assay. Granulocyte-erythrocyte-macrophage-megakaryocyte (GEMM), Granulocyte-macrophage (CFU-GM), Granulocyte (CFU-G), Macrophage (CFU-M), Pre-B cell (Pre-B), and Burst forming unit-erythroid (BFU-E). Magnification 400X.

 Hematopoiesis is characterized by a hierarchy of cells, with hematopoietic stem cells (HSC) possessing the highest proliferative potential and thought to be the targets of aberrant interchromosomal DSB repair events leading to mutagenic chromosomal rearrangements. Our previous *in vitro* studies demonstrated that early stem and progenitor cells are more proficient than terminally differentiated myeloid cells in repairing DSBs by interchromosomal HR [68]. Here, we determined the potential for hematopoietic multi-lineage progenitor cells to utilize this mechanism of repair *in vivo*. GS mice (ages 3-5 months) were administered tetracycline, then bone marrow cells harvested, and subsequently seeded into methylcellulose colony forming assays that support proliferation of myeloid, erythroid, or B-cell progenitors [68–70]. Total numbers of hematopoietic CFUs were scored and classified based on their morphology, and individual GFP+ CFUs determined by inverted fluorescent microscopy (Figure 7). Mature colonies derived from individual precursors included the colony forming unit- granulocyte-erythrocyte-monocyte-megakaryocte (CFU-GEMM), granulocyte-monocyte (CFU-GM), granulocyte (CFU-G), monocyte (CFU-M), erythrocyte (CFU-E), and pre-B (CFU-pre-B). Colonies that contain mixed cell populations are presumed to derive from immature progenitor cells capable of differentiation into multiple cell types. Colonies that contain a single cell population are presumed to derive from more differentiated progenitors that only have the capacity to expand a single cell type. 

 Following DSBs, GFP+ recombinants were readily obtained from all sub-populations assayed. Strikingly, the results parallel observations previously made in studies of DSB-induced interchromosomal HR using genetically engineered murine ES cells differentiated *in vitro* into hematopoietic colonies [68]. The highest average number of GFP+ recombinant colonies (32 ± 15) was observed in the multi-potent CFU-GEMM cells scored by this assay (Table 1). Observed numbers of GFP+ recombinants decreased with increased differentiation status with the lowest average number of GFP+ recombinant colonies (5 ± 5) observed in the terminally differentiated but actively proliferating monocytic cells (p value = 0.02) (Table 1). The average frequency of recombination in this *in vivo* system was estimated to be 8.0 x 10^-5^ in CFU-GEMM cells, 5.5 x 10^-5^ in CFU-GM cells, 6.5 x 10^-5^ in CFU-G cells, and decreasing to 1.25 x 10^-5^ in CFU-M cells. Overall these data demonstrate that both multipotent and terminally differentiated cell types retain the potential to repair DSBs with a homologous sequence on a heterologous chromosome *in vivo*.

**Table 1:** DSB-induced interchromosomal HR in hematopoietic progenitor cell populations.

		# GFP colonies**^*a*^**		
**Bone Marrow CFC**	**Expt 1**	**Expt 2**	**Expt 3**	**avg. GFP colonies**
CFU-GEMM	47	17	32	32 ± 15
CFU-GM	31	0	35	22 ± 19
CFU-G	23	49	7	26 ± 21
CFU-M	0	4	10	5 ± 5 p value = 0.02**^*b*^**
CFU-Pre B	37	21	27	28 ± 8
BFU-E	8	0	40	16 ± 21

GS mice were administered tetracycline via drinking H_2_O for 14d. Mice were then sacrificed, and femur bone marrow cells isolated and seeded into methylcellulose colony forming assays. Cells were plated at 1.0 x 10^5^ cells/plate. Each experiment included 4 technical replicates and the total number of colonies is shown.

*a* GFP+ colony numbers were normalized to account for variation of overall plating efficiency (total number of CFC of each type) between mice.

*b* Number of GFP+ CFU-M colonies observed was statistically significantly lower as compared to the number of GFP+ CFU-GEMM observed (student’s T test).

 Because CFU represent clonal populations, the recombinant HR repair products could be verified at the sequence level. DNA was extracted from individual BM colonies, and nested PCR used to amplify across the two I-*Sce*I endonuclease DSB repair sites (Figure 1A). Because the 3’ UTR ends of the transgene sequences are unique, primers could selectively amplify each of the two transgenes (Figure 1A). A total of 22 individual BM colony PCR products were cloned and sequenced. Because each transgene is inserted in multiple copies, PCR will amplify both GFP+ recombinant and parental non-recombinant copies of the transgenes. These were distinguishable following TA cloning and sequencing of multiple TA clones from each BM colony PCR. This analysis verified that all 22 BM colonies contained a repaired GFP+ wild-type sequence on at least one allele. In 6 of 22 colonies this analysis detected HR repair at only one allele (4 at 1S-GFP and 2 at 2S-GFP). In 16 of 22 colonies this analysis detected HR repair at both alleles; however given the multiple copy inserts, these likely represented independent events. 

## Discussion

This study presents an *in vivo* model that directly demonstrates that DSB-induced interchromosomal HR occurs at readily detectable rates. GFP+ recombinant cells were readily detectable in a broad range of somatic cell types. Variability in numbers of GFP+ recombinant cells was observed between the multiple somatic cell types and mice in all cohorts examined. Such variability could be due to differences in GFP expression, recombination rates, clonal expansion of individual GFP+ recombinants, or I-*Sce*I transgene induction, expression, or stability. This mouse model initiates I-*Sce*I expression *in vivo* using a single bicistronic TET-ON system [65]. The experiments in MEFs with this system show strong and specific induction, but *in vivo* kinetics could be different. In addition, individual mice self regulate feeding and thus vary dosage to tetracycline. However, similar inter-mouse variability in the *in vivo* mouse model of spontaneous intrachromosomal/sister chromatid HR suggests that I-*Sce*I is not the major determinant of these results [30].

 Intrachromosomal HR may occur if homologous repeat sequences lie on the same chromosome in the same direct repeat orientation such as repetitive elements within several kb of each other. Several studies have used *Arabidopsis* and *N. tabacum* models to detect spontaneous and DSB-induced sister chromatid and intrachromsomal HR with spontaneous frequencies estimated at 10^-5^ to 10^-4^ [29,35,71,72] and up to 10,000X stimulation by I-*Sce*I expression [29,35]. Further, similar to *in vitro* findings, SSA was a predominant mode of DSB repair with ectopic joining contributing to a smaller subset of repair events [35]. In mice, spontaneous intrachromosomal and sister chromatid HR have been demonstrated utilizing a yellow fluorescent protein (YFP) reporter or LacZ/β-galactosidase reporter construct [27,30,73–76]. These studies demonstrated median spontaneous HR frequencies of 5 per 10^6^ cells in the pancreas [30,73–76]. Ionizing radiation or the interstitial cross-linking agent mitomycin-C led to an increase of recombination suggesting that non-specific DNA damage is also sufficient to promote intrachromsomal HR, at least in pancreatic cells [75]. Although comparisons between different model systems are difficult, these results are surprisingly similar to the findings presented here suggesting that both types of HR repair are utilized with roughly the same overall efficiency, although likely in different cell types or at different stages of the cell cycle [60].

Cytologic studies indicate that nuclei are ordered, and chromosomes/genomes generally exist within defined nuclear territories [77,78], and single DSBs remain stable in these defined regions [79,80]. Genetic studies seem to support this model as repair of a single DSB in mouse and human cells does not promote large scale genome rearrangements between heterologs, although they can be associated with regional loss of heterozygosity (LOH) and insertions with sequences of unknown origin [20,31–33,81]. Similarly, multiple DSBs on the same chromosome do not significantly promote large-scale genome rearrangements in mouse or human cell lines, although the efficiency of repair decreases as the distance between two DSBs increases (up to 9 kb apart) [82,83]. 

By contrast, cytological analysis indicates chromosome movement is more fluid in DNA repair deficient cells [79]. Chromosome movement has also been observed in the presence of multiple induced DSBs on heterologous chromosomes in mitotic yeast [84] or following global exposure of cells to ionizing radiation or topo II inhibitors [85–87], leading to foci suggestive of repair centers (“repairosomes” [84,88]). The steps by which such repairosomes are initiated by chromatin remodeling programs as a normal step in DNA repair or the biological understanding of how translocations are formed within the ordered nucleus remain unclear [2,89,90]. It is not clear if chromosome movement is in response to multiple breaks in different loci or after prolonged or persistent damage. The established nuclear matrix and chromatin loop structures may also influence choice of recombination partners during DSB repair [89]. In support of the cytologic data, our genetic study here indicates that *in vivo* interaction of DNA sequences and recombination is promoted by multiple DSBs. 

A wider range of HR mechanisms are used to repair DSBs on heterologous chromosomes as compared to intrachromosomal HR. In addition, intrachromosomal HR is not typically associated with the genome rearrangements observed in human tumors. DSBs in cultured ES cells and multiple *in vitro* differentiated hematopoietic cell types can stimulate interchromosomal HR as a repair pathway [68]. We observed with this *in vivo* system that repair by HR in multiple hematopoietic lineages is also quite prevalent with observed GFP+ numbers decreasing with differentiation. Similarly, in most organs the number of GFP+ cells decreased with age. These data support other hypotheses that differentiation and age will determine different pathways of repair or utilize apoptotic programs with different frequencies [57–60].

Topo II is an essential cellular enzyme that catalyzes changes in DNA topology via its cleavage-religation equilibrium. Topo II inhibitors convert topo II into a DNA-damaging enzyme by disrupting the cleavage-religation equilibrium, resulting in accumulation of DSBs, activation of DNA damage sensors, cell cycle arrest, and initiation of apoptosis or repair. A wide range of agents, including some chemotherapeutic agents, are classified as topo II inhibitors, and exposure to these is associated with development of secondary leukemias [91,92]. However, they also include benzene metabolites, bioflavinoids, anthraquinone laxatives, podophyllin resins, quinolone antibiotics, pesticides, many phenolic compounds, as well as certain fruits, tea, coffee, wine, soy, and cocoa [11,12,93]. The recent observations that bioflavinoids can stabilize DNA DSBs and promote illegitimate repair and genome rearrangements in cultured cells has led to the hypothesis that exposure to these agents *in utero* or through unregulated high doses as dietary supplements may promote leukemia [15–18]. Further study of this *in vivo* system could determine the potential for exposure to such agents at early stages of development to promote HR *in vivo* and their long-term impact.

Rearrangements resulting from DSB repair that occurs in germ cells can have evolutionary implications. It has been observed that topoII has a role in DSB formation in spermatids [94], and chromatin loop organization is similar between spermatids and somatic cell types [89]. These observations have led to the suggestion that DSB repair pathways and partner choice may be more similar in meiotic and mitotic cells than appreciated and has the potential to result in rearrangements leading to genome variation [89,95]. That this may be universal across multiple kingdoms, is supported by genome analysis of plants that suggests translocations are a regular mechanism of plant evolution [40,41]. In addition, mutation fixation has been implicated during DSB repair in the first zygotic cell division in mice [96]. Our demonstration that interchromosomal HR occurs *in vivo* in response to DSBs at just two loci in a broad range of cell types, particularly progenitor cells, is a novel finding and lends further support to the idea that exposure to the growing list of environmental agents, dietary supplements, or groundwater contaminants that induce or stabilize DSBs may promote potentially tumorigenic rearrangements, accelerate genomic variation, and influence evolution.

## Materials and Methods

### Ethics Statement

All studies were approved by IACUC (protocol #AAAA0123 Columbia University; protocol #08-035 University of North Carolina at Charlotte). All studies were conducted under supervision of appropriate regulatory bodies and in accordance to established NIH guidelines for ethical treatment of animals in research.

### Transgenic Mice

Transgenic mice for study were generated by establishing three independent transgenic lines of mice: (1) the tetracycline-regulated I-*Sce*I expression gene, (2) 1SGFP with I-*Sce*I cut site 1, and (3) 2SGFP containing I-SceI cut site 2 (1). TET-I-*Sce*I – XbaI-PstI fragment of CBAS containing the I-*Sce*I gene [64,66] sequence was inserted into NheI-BamHI digested pBIG3i bicistronic tetracycline-regulated vector (kindly provided by Craig Strathdee) [65]. DNA was digested with BspHI and the fragment was provided to the Columbia University Transgenic Mouse facility (2). 1SGFP – SacII-HindIII fragment of pCAGGS-NZE-GFP containing the GFP sequence was sub-cloned into SacII-HindIII digested pBluescript SK+, creating SKRGFP(Sac2H3). Single-stranded oligomers BHI-∆1-ISceI-NcoI P1 (5’-GATCTGGATCCACCGGTCGCAATTACCCTGTTATCCCTACCATGGAGTAC-3’) and BHI-∆1-ISceI-NcoI P2 (5’-GTACTCCATGGTAGGGATAACAGGGTAATTGCGACCGGTGGATCCAGATC-3’) were annealed and digested with BamHI and NcoI. This BamHI-NcoI fragment containing the I-SceI recognition site was ligated into the BamHI-NcoI digested SKRGFP, creating SKRGFP(Sac2H3)∆1-S. The SacII-HindIII fragment of SKRGFP(Sac2H3)∆1-S was subcloned back into the SacII-HindIII digested pCAGGS-NZE-GFP plasmid, to create pCAGGS-GFP∆1-S. DNA was digested with SalI and PstI, and the 3433 bp fragment was provided to the Columbia University Transgenic Mouse facility (3). 2SGFP -- A PvuII site was engineered in pCAGGS-NZE-GFP using annealed single-stranded oligomers GFP-Pvu2-1 (5’-CGCCGACCACTACCAGCTGAACACCCCCATCGGCGAC-3’) and GFP-Pvu2-2 (5’-GTCGCCGATGGGGGTGTTCAGCTGGTAGTGGTCGGCG-3’and QuikChangeII Site-Directed Mutagenesis Kit (Stratagene), following manufacturer’s protocol, creating pGFP-Pvu2. Single-stranded oligomers SCE1 (5’-Phos-ATTACCCTGTTATCCCTA)and SCE2 (5’-Phos-TAGGGATAACAGGGTAAT-3’) were annealed and ligated into the PvuII blunt end digested pGFP-Pvu2, creating pGFP-Pvu2-S. DNA was digested with SalI and PstI, and the 3433 bp fragment was provided to the Columbia University Transgenic Mouse facility. The Columbia University Transgenic Mouse facility generated transgenic mice in F1 (C57BL/6J-CBA) hybrids, and mice were transferred to University of North Carolina at Charlotte. 

The two GFP lines were intercrossed and the resulting line crossed with mice containing the tetracycline regulated I-SceI expression transgene. The resultant triply positive transgenic line was denoted “GS” and used for further study. Genotyping for presence of all three transgenes was performed by PCR and Southern blotting of mouse tail tip genomic DNA and subsequent digestion of PCR products with I-*Sce*I endonuclease (New England Biolabs) to confirm intact I-*Sce*I sites. Amplification was performed by 94°C 5 min; followed by 40 cycles of 94°C 30s, 60°C 30s, 72°C 2 min; and 72°C 15 min. For nested PCR 5uL of the first PCR product was used as template for second round of PCR following the same protocol. PCR primers for each: Sce1F 5’-gtccgaactctaaactgctga-3’; Sce2R 5’-ACCAGTATGCCAGAGACATC-3’; GFP 1F 5’-aaggccaagagggccaa-3’; GFP 2F 5’-TGGACGGCGACGTAAAC-3’; GFP 3R 5’-gtgctcaggtagtggttg-3’; GFP 4R 5-CTCTGTTCCACATACACTTC-3’; GFP 5F 5’-tgaaccgcatcgagctgag-3’; GFP 6R 5’-GACCATGTGATCGCGTTC-3’; GFP 7R 5’-TTCTGATAGGCAGCCTG-3’. Southern blotting to determine copy number utilized a plasmid fragment of full length GFP ORF of 3.07 kb and diluted to pg amounts that approximated 0.2, 1.0, 5.0, 10, 20, and 100 copies per genome spiked into 10µg non-transgenic mouse DNA. Genomic DNA of transgenic mice was digested with restriction endonucleases that flank the GFP promoter and ORF of both transgenes. The GFP probe fragment was an Sph-Not I fragment homologous to both transgenes. Q-PCR for copy number estimation utilized a GFP ORF fragment diluted to pg amounts that approximated 0.2, 1.0, 5.0, 10, 20, and 100 copies per genome to amplify a 296 bp fragment of GFP DNA. Genomic DNA isolated from transgenic mice was utilized for Q-PCR.  Fluorescent detection of PCR products was reported using a SYBR® Green PCR kit (Quanti Tect) in 20μL reactions established according to the manufacturer’s recommended protocol.  A standard curve was generated (n=3) using the control plasmid GFP ORF DNA according to the manufacturer’s protocol (QuantiTect). Q-PCR analysis was simultaneously analyzed by a 96 well 7500 Fast Real-Time PCR System (Applied Biosystems) in which transgenic mouse genomic DNA was compared against the standard curve and statistical analysis performed according to the Applied Biosystems protocol for 7500 Fast Real-Time PCR System protocol.

### MEFs

 Mouse embryonic fibroblasts were isolated from day E13.5 of GS mice and washed with phosphate buffered saline (PBS). The head was removed from isolated embryos and used for DNA genotyping. The body was minced well and 10mL of 0.25% Trypsin-EDTA (Gibco, Grand Island, NY) added. Solution was triturated with a pipette and added to 25 mL of medium [Dulbecco’s Modified Eagle Medium (Gibco), 15% FBS (Gemini Bio-Products, West Sacramento, CA), 1.2% 200mM L-Glutamine (Gemini Bio-Products), 1.2% Non-essential Amino Acids (Gibco), and 1.2% Penicillin-Streptomycin (Gibco)]. Cells were then collected by centrifugation (1000 rpm x 10 min), resuspended into 4mL medium, and cultured on a 6-well dish at 37°C with 5%CO_2_. MEFs were then passaged onto 10cm dishes after initial growth. Tetracycline HCl (Barr Laboratories) was dissolved in 1x PBS to 1mg/mL and passed through a 0.2 micron filter. MEFs were given a final concentration of 2μg/mL for up to 6 days. 

### DSB induction in Mice

 Tetracycline HCl (Barr Laboratories) was dissolved into .5X PBS/H20/ sucrose at 10mg/mL and passed through a 0.2 micron filter. Mice were administered tetracycline at 2mg/mL in a water bottle for up to 21 days. 

### Flow cytometry and statistical analysis

MEFs were trypsinized and collected by centrifugation (1000 rpm x 10 min). Cells were resuspended in 1x PBS at a concentration of 1.0x10^6^ cells/mL. Sections of individual organs were harvested and a single cell suspension generated in 5% Bovine Serum Albumin (Gemini Bio-Products)/1x PBS. Suspensions were passed through a 53μM nylon mesh filter (Spectrum Laboratories Inc) and analyzed on a FACSAriaII for GFP positivity. To assess statistical significance of increased numbers of GFP+ cells among organs in tetracycline treated mice (Table S1), we utilized a non-parametric t-test for all mice versus negative controls (Figure 5), and then separately in the young and old cohorts versus negative controls (Figure 6). To assess statistical significance of the probabilities associated with covariance of the number of GFP+ cells in organs of individual mice, we calculated Spearman’s nonparametric correlation coefficients for each pair of organs separately in the young and old cohorts, and utilized the false discovery rate procedure to control the proportion of false positive results (Table S2). 

### Western Immunoblot analysis

Protein was isolated from pelleted cells using Total Protein Extraction Kit (Millipore). Cell lysate proteins were then separated on a 10% NuPage Bis-Tris SDS-Page gel (Invitrogen) and transferred to Amersham Hybond-P membrane (GE Healthcare Life Sciences). The membranes were then blocked in 5% Non-Fat dry milk in 1X tris buffered saline (Bio-Rad). Membranes were probed with a mouse monoclonal IgG anti-HA antibody to detect the HA tag within I-*Sce*I (Cell Signaling Technology) at 1:100 dilutions for 20-22 hours at 4°C or a mouse monoclonal IgG anti-β-actin antibody (Santa Cruz Biotechnology) at 1:400 dilution for 1 hour at room temperature. Blots were subsequently exposed to an anti-mouse IgG HRP-linked secondary antibody (Cell Signaling Technology) at 1:1000 dilutions for 1 hour at room temperature. Blots were washed 3x for five minutes each in a 1x TBS-.05% Tween 20 solution. Membranes were developed using SuperSignal® West Pico Chemiluminescent Substrate (Thermo Scientific).

### Bone Marrow-CFC Assay

GS mice (ages 3-5 months) were administered tetracycline through H_2_O for 14d. Mice were then sacrificed, and femur bone marrow (BM) cells isolated and seeded into methylcellulose colony forming assays [69,70]. Whole BM was flushed from femurs into IMDM supplemented with 2% FBS and disrupted into a single cell suspension by a 22G needle and syringe. Cell viability counts were performed using .05% trypan blue staining. Total viable BM cells were plated at 1.0 x 10^5^ cells per 35mm low adherence tissue culture dishes in hematopoietic differentiation medium (STEMCELL Technologies) containing IMDM, 1% methylcellulose, 15% non-ES qualified FBS, 100U/mL penicillin, 100μg/mL streptomycin, 2mM L-glutamine, 150μM monothioglycerol, 1% bovine serum albumin, 10μg/mL insulin, 200μg/mL transferrin, 150ng/mL mSCF, 30ng/ml mIL-3, 30ng/mL mIL-6, and 3U/ml hEPO for 14 days. 

### DNA Sequence Analysis of HR Recombinants from BM CFCs

Individual CFU-GEMM expressing GFP were identified by inverted fluorescent microscopy and isolated. Genomic DNA from 24 individual CFU-GEMMs was extracted from each with DNeasy Tissue Kit (Qiagen) followed by whole genome amplification (WGA) with Repli-G Kit (Qiagen) as previously described [68]. 1.0μg of WGA DNA template was used for PCR. Each 25μL PCR reaction contained template DNA, 10X reaction buffer, 1.5mM MgCl_2_, 200μM each dNTP, 0.48μM each primer, 2.5 units Taq DNA polymerase. PCR primer sets are indicated in Figure 1 and in Methods above. Amplification was performed by 94°C 5 min; followed by 40 cycles of 94°C 30s, 55°C 30s, 72°C 2 min; and 72°C 15 min. For nested PCR 5uL of the first PCR product was used as template for second round of PCR following the same protocol. PCR reaction products were cloned with the TA cloning system (Invitrogen) and blue-white screening used to determine which individual clones to amplify, isolate DNA, and sequence by Sequetech (Mountain View, CA) using M13 forward and M13 reverse primers. Sequencing of up to 10 white colonies from each PCR reaction/TA cloning reaction was sufficient to identify GFP+ recombinants among parental GFP sequences. 

## Supporting Information

Table S1 **Number of GFP+ cells detected per million analyzed by FACS in young and old cohorts.** Individual mice are noted with young cohort mice indicated by Y and old cohort mice indicated by O. Organs from which technical error led to no sample recovered for FACS analysis are noted as nd (no data). These values were the basis for the covariance of traits analysis in Table S2.
(DOCX)

Table S2 **Covariance of GFP+ cells in organs of young and old cohorts.** To assess statistical significance of the probabilities associated with covariance, Spearman’s nonparametric correlation coefficients for each pair of traits separately in the young and old cohorts (Table S1), and utilized the false discovery rate procedure to control the proportion of false positive results. Calculated p-values in the young mouse cohort are represented within the top diagonal half of the matrix. Calculated p-values in the old mouse cohort are represented within the bottom diagonal half of the matrix. p-values <0.05 are denoted with **.
(DOCX)
