# Supplementary material for: Double-Strand Break Repair by Interchromosomal Recombination: An In Vivo Repair Mechanism Utilized by Multiple Somatic Tissues in Mammals
Source: PLoS One. 2013 Dec 13;8(12):e84379. doi: 10.1371/journal.pone.0084379 (PMC3862804; doi:10.1371/journal.pone.0084379)
Supplement: Table S1 — Number of GFP+ cells detected per million analyzed by FACS in young and old cohorts. Individual mice are noted with young cohort mice indicated by Y and old cohort mice indicated by O. Organs from which technical error led to no sample recovered for FACS analysis are noted as nd (no data). These values were the basis for the covariance of traits analysis in Table S2. (DOCX) [file pone.0084379.s001.docx]

**Table S1. Number of GFP+ cells detected per million analyzed by FACS in young and old cohorts.** Individual mice are noted with young cohort mice indicated by Y and old cohort mice indicated by O. Organs from which technical error led to no sample recovered for FACS analysis are noted as nd (no data). These values were the basis for the covariance of traits analysis in Table S2.

| ID | Heart | Pancreas | Liver | Kidney | Spleen | Lung | Thymus |
| --- | --- | --- | --- | --- | --- | --- | --- |
| Y102 | 233 | 381 | 29 | 225 | 81 | 234 | nd |
| Y104 | nd | 67 | 71 | 31 | 11 | nd | nd |
| Y105 | nd | 7 | 9 | 9 | 5 | nd | nd |
| Y100 | 5 | 2 | 22 | 22 | 18 | 7 | 49 |
| Y142 | 0 | 47 | 83 | 4 | 5 | 1 | 30 |
| Y150 | 0 | 25 | 34 | 4 | 1 | 10 | 81 |
| Y154 | 0 | 2 | 29 | 5 | 5 | 3 | 3 |
| Y155 | 1 | 2 | 21 | 0 | 0 | 9 | 5 |
| Y158 | 4 | 2 | 5 | 4 | 4 | 1 | 84 |
| Y157 | 6 | 2 | 1 | 1 | 1 | 91 | nd |
| Y156 | 0 | 70 | 16 | 65 | 65 | 32 | nd |
| Y179 | 10 | 0 | 3 | 17 | 17 | 10 | 40 |
| Y182 | 35 | 17 | 0 | 28 | 27 | 37 | 0 |
| Y180 | 20 | 19 | 0 | 27 | 27 | 10 | 10 |
| Y183 | 12 | 3 | 0 | 5 | 5 | 0 | 141 |
| Y181 | 4 | 0 | 0 | 0 | 0 | 8 | 33 |
| O108 | 96 | 5 | 344 | 2 | 11 | 1 | 4 |
| O109 | 570 | 6 | 108 | 3 | 5 | 4 | 5 |
| O110 | 11 | 2 | 2 | 6 | 6 | 77 | 190 |
| O111 | 16 | 2 | 0 | 215 | 17 | 39 | 63 |
| O96 | 1 | 2 | 0 | 10 | 4 | 1 | 2 |
| O98 | 12 | 1 | 0 | 3 | 0 | 2 | 0 |
| O123 | 30 | 7 | 3 | 12 | 28 | 4 | 79 |
| O125 | 76 | 2 | 5 | 7 | 188 | 0 | 68 |
| O130 | 7 | 3 | 5 | 2 | 5 | 4 | 15 |
| O99 | 7 | 12 | 5 | 7 | 16 | 9 | 44 |
| O113 | 0 | 0 | 19 | 8 | 7 | 4 | 2 |
| O114 | 2 | 1 | 7 | 4 | 3 | 3 | 0 |
| O118 | 0 | 0 | 15 | 17 | 2 | 0 | 7 |
| O117 | 13 | 1 | 43 | 5 | 5 | 10 | 2 |
| O116 | 0 | 1 | 24 | 6 | 6 | 12 | 0 |
| O115 | 1 | 0 | 53 | 3 | 6 | 1 | 0 |
| O101 | nd | 4 | 124 | 10 | 9 | nd | nd |
| O141 | 3 | 13 | 1 | 5 | 3 | 6 | 1 |
| O145 | 1 | 1 | 3 | 5 | 1 | 17 | 3 |
| O171 | 6 | 6 | 1 | 9 | 0 | 7 | 0 |
| O165 | 0 | 0 | 0 | 6 | 7 | 11 | 2 |
| O139 | 1 | 13 | 1 | 2 | 2 | 5 | 17 |
| O137 | 9 | 74 | 2 | 2 | 2 | 10 | 2 |
| O121 | 11 | 50 | 159 | 1 | 1 | 5 | 42 |
| O161 | 0 | 2 | 0 | 3 | 0 | 0 | 0 |
| O148 | 0 | 1 | 0 | 2 | 0 | 0 | 3 |
| O170 | 0 | 0 | 0 | 2 | 1 | 3 | 0 |
| O167 | 1 | 0 | 0 | 3 | 4 | 1 | 3 |
| O144 | 1 | 1 | 0 | 3 | 3 | 0 | 1 |
| O166 | 0 | 2 | 0 | 2 | 2 | 2 | 0 |
| O153 | 0 | 3 | 0 | 0 | 0 | 4 | 0 |
|  |  |  |  |  |  |  |  |
