# Supplementary material for: Double-Strand Break Repair by Interchromosomal Recombination: An In Vivo Repair Mechanism Utilized by Multiple Somatic Tissues in Mammals
Source: PLoS One. 2013 Dec 13;8(12):e84379. doi: 10.1371/journal.pone.0084379 (PMC3862804; doi:10.1371/journal.pone.0084379)
Supplement: Table S2 — Covariance of GFP+ cells in organs of young and old cohorts. To assess statistical significance of the probabilities associated with covariance, Spearman’s nonparametric correlation coefficients for each pair of traits separately in the young and old cohorts (Table S1), and utilized the false discovery rate procedure to control the proportion of false positive results. Calculated p-values in the young mouse cohort are represented within the top diagonal half of the matrix. Calculated p-values in the old mouse cohort are represented within the bottom diagonal half of the matrix. p-values <0.05 are denoted with **. (DOCX) [file pone.0084379.s002.docx]

**Table S2. Covariance of GFP+ cells in organs of young and old cohorts.** To assess statistical significance of the probabilities associated with covariance, Spearman’s nonparametric correlation coefficients for each pair of traits separately in the young and old cohorts (Table S1), and utilized the false discovery rate procedure to control the proportion of false positive results. Calculated p-values in the young mouse cohort are represented within the top diagonal half of the matrix. Calculated p-values in the old mouse cohort are represented within the bottom diagonal half of the matrix. p-values <0.05 are denoted with **.

|  | Heart | Pancreas | Liver | Kidney | Spleen | Lung | Thymus |
| --- | --- | --- | --- | --- | --- | --- | --- |
| Heart |  | 0.960 | 0.158 | 0.317 | 0.317 | 0.342 | 0.960 |
| Pancreas | 0.021** |  | 0.317 | 0.110 | 0.158 | 0.517 | 0.960 |
| Liver | 0.074 | 0.529 |  | 0.960 | 0.960 | 0.960 | 0.960 |
| Kidney | 0.695 | 0.587 | 0.769 |  | 0.002** | 0.342 | 0.797 |
| Spleen | 0.074 | 0.947 | 0.074 | 0.021** |  | 0.342 | 0.744 |
| Lung | 0.587 | 0.502 | 0.700 | 0.696 | 0.587 |  | 0.342 |
| Thymus | 0.021** | 0.175 | 0.320 | 0.417 | 0.021** | 0.587 |  |
